# Morphological analysis of anterior permanent dentition in a Chinese population using cone-beam computed tomography

**DOI:** 10.1186/s13005-023-00357-2

**Published:** 2023-03-24

**Authors:** Yu Chen, Yu Dai, Zhengbin Yan, Yuehua You, Bin Wu, Bingtai Lu

**Affiliations:** 1 Department of Dentistry, People’s Hospital of Longhua, Shenzhen, 518109 Guangdong China; 2 Department of Dentistry, Shenzhen People’s Hospital, Shenzhen, 518020 Guangdong China; 3 Medical Research Institute, Guangdong Provincial People’s Hospital (Guangdong Academy of Medical Sciences), Southern Medical University, Guangdong 510080 Guangzhou, China

**Keywords:** Cementoenamel junction curvature, Cone-beam computed tomography, Furcation, Root canal, Root length

## Abstract

**Purpose:**

Morphological analysis of permanent anterior dentition is essential for achieving an ideal treatment outcome and avoiding unnecessary failure. This study aimed to analyze the morphologies of anterior teeth in the Chinese population in depth.

**Methods:**

In this retrospective study, 4309 anterior teeth from 401 Chinese patients were investigated using cone-beam computed tomography (CBCT) from 2019–2021. We summarized the morphological characteristics of the anterior teeth in terms of the root length, cementoenamel junction curvature (CEJ-C), root furcation and canal variations.

**Results:**

We found that the root lengths of the maxillary anterior incisors were similar (13.3 mm), while the root lengths of the mandibular central (12.2 mm) and lateral incisors (13.4 mm) varied significantly (*p* < .0001). Both the maxillary (16.6 mm) and mandibular canines (15.5 mm) were found to have greater root lengths than the corresponding incisors (*p* < .0001). The CEJ-C was significantly greater around incisors (2.5 mm) than around the canines (2.0 mm) in the maxilla (*p* < .0001), while the curvature remained similar in mandibular anterior teeth (1.8 mm). Root furcation was observed in mandibular canines and lateral incisors. Moreover, all types of Vertucci’s classification in anterior dentitions were observed, while two other new types were found. Among them, the maxilla was only observed to exhibit types I, II, III, and ST II, while the mandible was found to exhibit almost all types. However, Type I still accounts for the majority of dentitions.

**Conclusions:**

Morphological analysis of permanent anterior dentition revealed diversity in the tooth length, CEJ-C, furcation proportion, and canal variations. In general, mandibular anterior teeth showed a more complex structure than maxillary teeth.

## Introduction

Root morphology is important for oral health and dental treatment, including but not limited to endodontic-treated tooth preservation and tooth extraction [1]. Although the general morphology of specific tooth positions is similar throughout all human races, they still vary in multiple aspects, such as the root length, root furcation, root canal classifications, and cementoenamel junction curvature (CEJ-C) [2]. Unlike molars, which are widely known to have complicated root morphology, anterior permanent dentition is always taken lightly and mistreated with a single root and canal [3]. Therefore, it is essential to analyze and summarize the nationwide morphological characteristics of anterior dentition.

In 1971, prior to the emergence of radiographic intervention, Skidmore & Bjorndal extracted 45 mandibular first molars and fabricated plastic casts of root canals out of the teeth for investigation [4]. Çalişkan et al. also utilized 1400 extracted teeth from Turkish patients to analyze the root canal morphology [5]. However, the results relied on indirect observation and were further limited by complex tooth processing procedures in vitro. Later, panoramic X-ray film and periapical film became available and offered a direct but opaque view of the anterior root canal due to reconstructive distortion and 2-D image overlap [6]. With the progress of medical imageology, cone-beam computed tomography (CBCT) was subsequently introduced to dental science. Unlike traditional methods, CBCT is a noninvasive method that conveniently and swiftly unveils microstructures. Therefore, an increasing amount of research has relied on this advanced technology to observe dental internal structures such as tooth canals or osseous lesions [7, 8]. Although previous articles have examined the root lengths and root canals, the CEJ-C has rarely been described.

Endodontic treatment failure or even tooth extraction is always attributed to nonstandard treatment procedures, while a limited acquaintance with tooth anatomical morphology is one of the obstructions [9]. As a gold standard for classifying root canal morphology, Vertucci sorted out eight types of root canals in permanent dentition in 1984, which was then named Vertucci’s classification. Later, this classification was adopted extensively in clinical analysis [10]. Some research groups have investigated specific tooth morphology using CBCT and have drawn conclusions based on Vertucci’s classification domestically; this research has been conducted in Malaysian [11], Brazilian [12], Iranian [13], and Chinese populations [14]. However, with the invention of CBCT, an increasing number of root canal types have been observed [1]. All these studies suggested fragments of root information among various races [15–17]; therefore, it is necessary to investigate and compare anterior dentition in the Chinese population.

Therefore, the current study aimed to provide a comprehensive blueprint of the morphological characteristics of anterior permanent dentition for the Chinese population using CBCT. Moreover, important factors such as root length, furcation, CEJ-C, and root canals should be investigated thoroughly.

## Materials and methods

This retrospective study was carried out in accordance with the Strengthening the Reporting of Observational Studies in Epidemiology (STROBES) guidelines [18, 19].

### Ethical approval and study samples

This retrospective study was approved by the ethics committee of People’s Hospital of Longhua, Shenzhen, China (2022–080). Informed consent was obtained verbally from all participants or their guardians. All CBCT scans were taken in the imaging department between 2019 and 2021.

The sample size was determined based on the single sample rate calculation formula: *n* = (Zα/δ)^2^⋅π(1 − π) [20, 21]. We took the maximal sample size at 95% confidence intervals with α = 0.05, δ = 0.05, where π is taken as 0.5 when π(1 − π) is at its maximum value of 0.25. Therefore, 269 samples are needed. To exclude sampling bias, 500 patients were recruited for this study, but 99 were excluded due to substandard CBCT scans or declining to participate. Ultimately, a total of 4309 teeth from 401 patients were included in this study. The CBCT images were not taken merely for this study but also for treatment purposes.

### Inclusion and exclusion criteria for CBCT images

The inclusion criteria for participants were as follows: (1) > 14 years old; (2) fully developed healthy permanent anterior teeth with completely formed root apex; and (3) good-quality CBCT images with high-resolution of lamina dura that were harvested in the past two years (2019–2021). The exclusion criteria were as follows: (1) teeth exhibiting wedge defects, orthodontic arch wires, crowns, fillings or analogs at the cervical area obscuring the cementoenamel junction (CEJ); and (2) roots with internal resorption, root canal filling, fracture, post and cores or analogs that conceal the root canal morphologies.

### CBCT specifications

Data were obtained from a CBCT machine (New Tom VG, New Tom machine, Bologna, Italy) with exposure parameters of 120 kVp, 3–7 mA, 26.9/8.9 s, a view field of 16.5 cm × 13.5 cm and an image resolution of 0.3 mm in this study. CBCT images were analyzed via NNT software (New Tom machine, Bologna, Italy). The CBCT images were viewed with an LED 25f display with a resolution of 1920 × 1080 pixels and a pixel pitch of 0.270 × 0.270 mm.

### Image interpretation and calibration

**Fig. 1:**
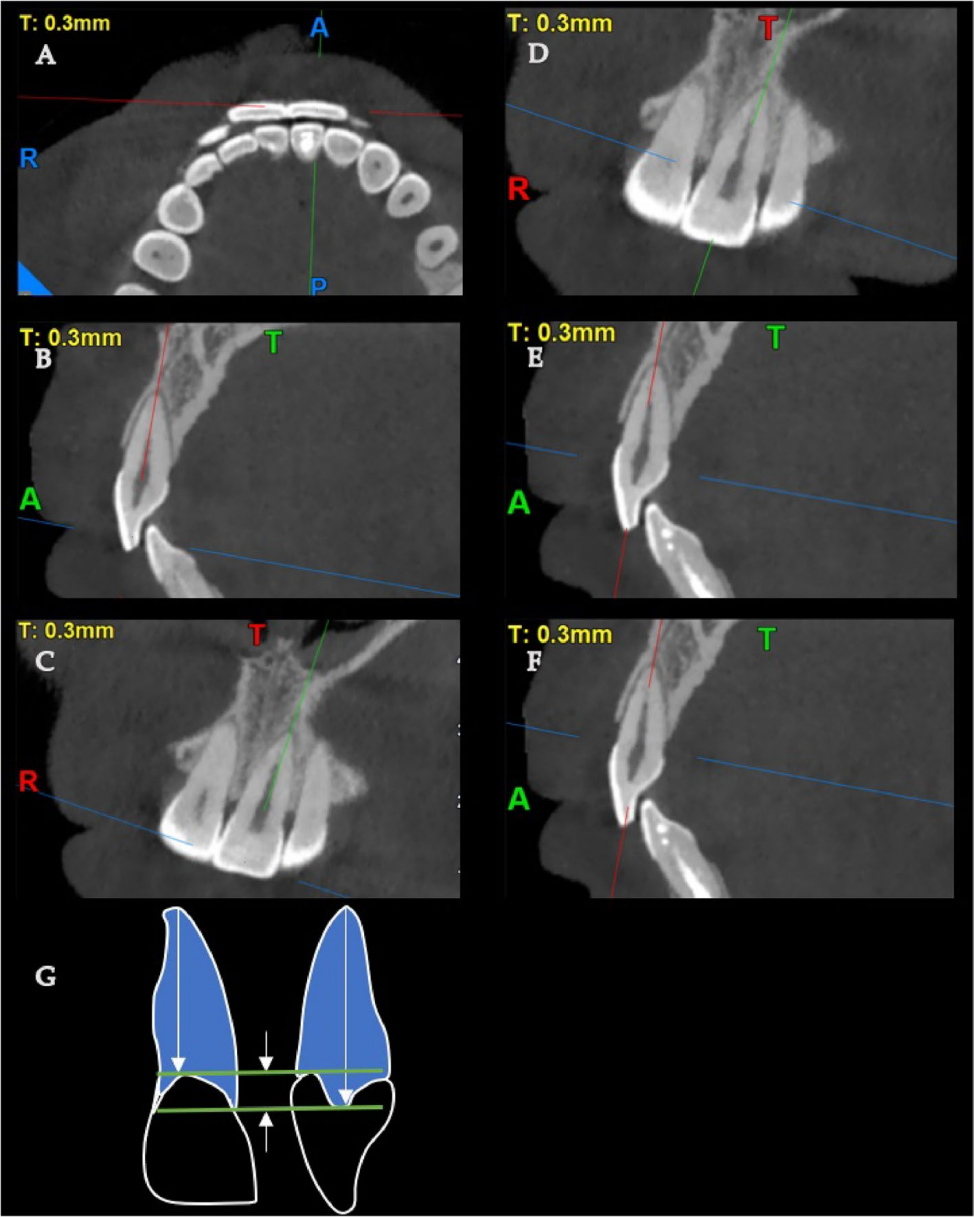
Determination of appropriate field of view. （**A**）Transverse plane; (**B**)(**E**)(**F**) Sagittal plane; (**C**)(**D**) Coronal plane; (**G**) Cementoenamel junction curvature. The red line represents the coronal plane, the green line represents the sagittal plane, and the blue line represents the transverse plane. First, the target tooth (maxillary left central incisor) is in sight, and we adjust the red line parallel to the incisal edge and pass through the central point of the tooth (**A**). Then, the angle of the red line can be adjusted subtly so that the red line fits into the center of the root canal, as shown in (**B**). At this time, the blue and green lines are shown in (**C**). While keeping the red line stable in position, we gently moved the blue line down to locate the cementoenamel junction (CEJ) in the mesial-distal direction coronally (**D**). At this time, the blue line was seen above the CEJ in the buccal-lingual direction in the sagittal plane (**E**). Finally, we adjusted the blue line apically to locate the CEJ in the buccal-lingual view in the sagittal plane (**F**). The root length was determined at both the buccal-lingual and mesial-distal views (**G**). The mean value was the final root length, and the subtraction was the CEJ-C value

Axial line determination is a crucial step in whole dentition analysis. Random selection of tooth angle and position in CBCT scans would lead to bias or even error. We could obtain accurate images by deliberately choosing appropriate planes passing through the tooth, and then the root length could be confirmed in a perpendicular field of view (Fig. 1A-F). Every tooth to be investigated would be analyzed in a step-by-step manner to ensure the accuracy of measurement.

Root lengths were calculated from the CEJ to the root apex at the buccal-lingual (BL) view and mesial-distal (MD) view. The average lengths of roots were calculated as the final root length. The method for determining the CEJ-C value is shown in Fig. 1G. Subtraction of root length from BL and MD views was obtained, representing the CEJ-C of every single tooth. Since the CEJ is not a straight curve but a wave with the vertex at the MD side and bottom at the BL side, the final CEJ-C value was also an averaged value representing the CEJ-C (Fig. 1G). The root canal morphology of maxillary and mandibular anterior teeth was analyzed according to Vertucci’s classification [10].

The observers evaluated the tooth morphology three-dimensionally, as shown in Fig. 1. Measurements were calibrated by two senior dentists trained by endodontic and radiological specialists by determining the appropriate starting and ending points of the CEJ and root apex as well as root canal shapes. Calibration for accuracy was modulated by repeated measurements to reduce bias. The two dentists read every length separately, and the mean value was then calculated if the readings were similar. Otherwise, the reading process was repeated until similar measurements were obtained. Any detection of reading bias was discussed and re-evaluated for a consensus.

### Statistical analysis

Statistical analysis was performed using SPSS 25 (IBM Corporation, New York, NY, USA). The data were tested for normality using the Kolmogorov–Smirnov test. The root length and CEJ-C were reported as the mean ± SD if the data were normally distributed or medians if the data were not normally distributed. When analyzing if there was any difference among the six teeth at the maxilla or mandible, normally distributed variables were compared using t-test or analysis of variance (ANOVA), while nonnormally distributed data were compared using the Kruskal‒Wallis H test. *p* < 0.05 was considered statistically significant.

## Results

**Fig. 2:**
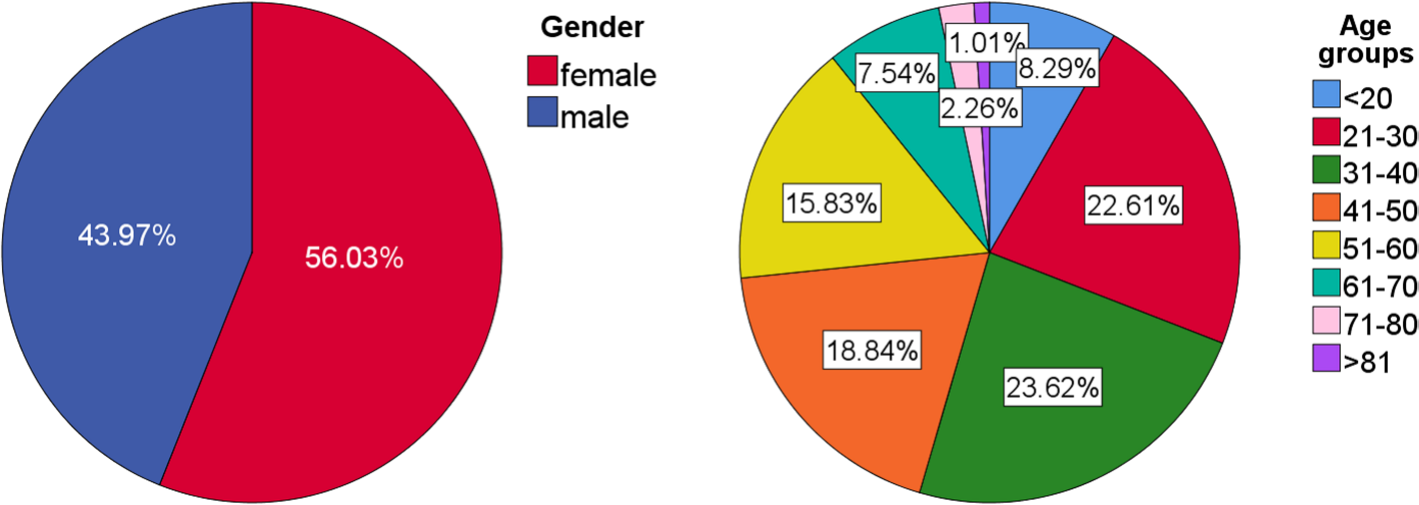
Demographic characteristics of participants with age and gender distributions

A total of 4309 teeth from 401 CBCT images (age 15–86 years, 223 females and 178 males) were included in this study (Fig. 2). Participants aged 21–60 accounted for the majority of the patients.

### Root and CEJ-C lengths of maxillary and mandibular anterior teeth

**Fig. 3:**
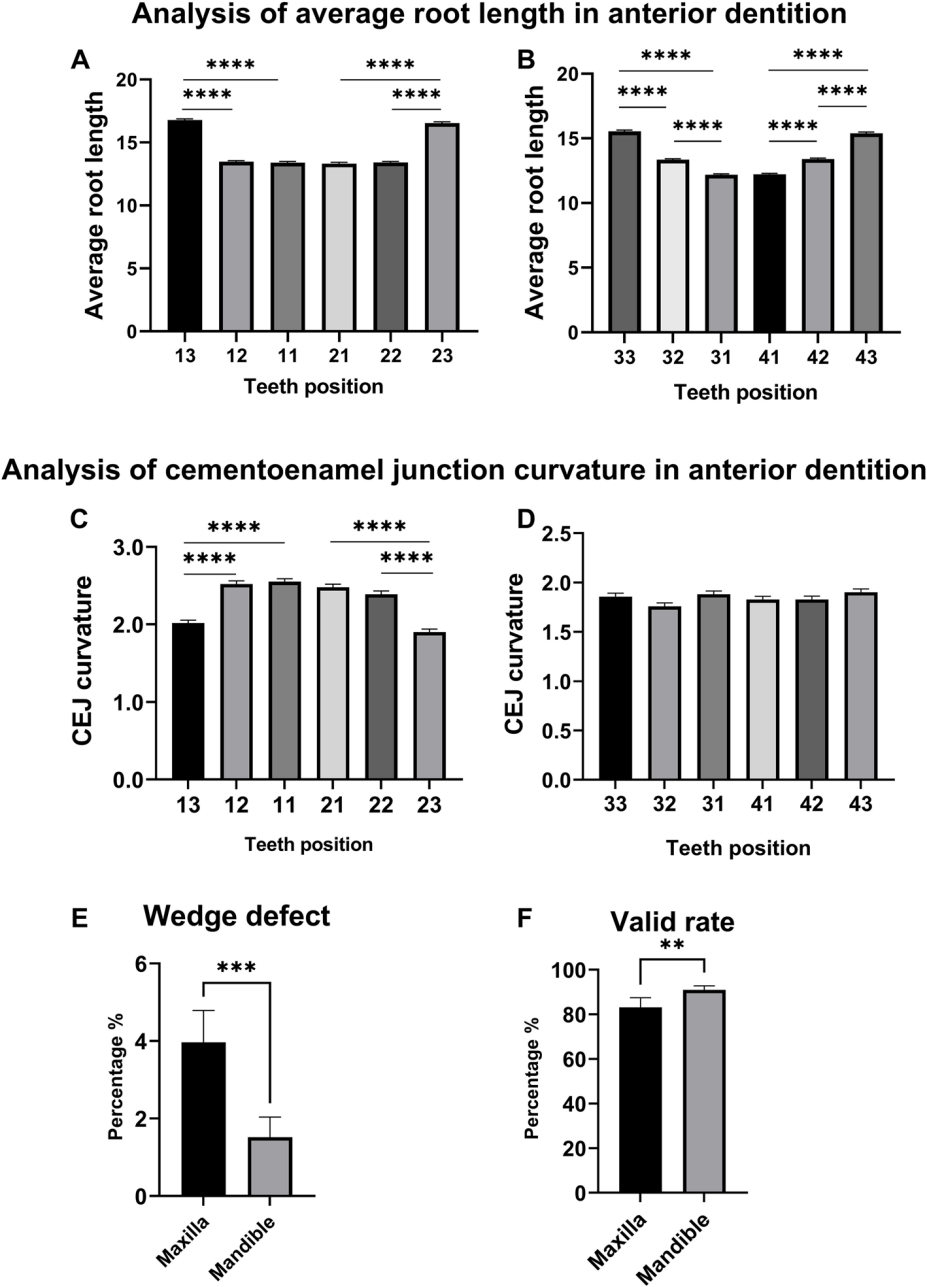
Analysis of various root indices in maxillary and mandibular teeth. Teeth position was expressed by the international/FDI tooth numbering system. **A-B** Average root length in maxillary and mandibular anterior teeth. One-way ANOVA was utilized for data analysis. **C-D** Differences in the CEJ-C values among maxillary and mandibular anterior teeth. The independent-samples Kruskal‒Wallis test was utilized for data analysis. **E–F** Analysis of wedge defects and valid rates of maxillary and mandibular anterior teeth. t-test was utilized for data analysis

Maxillary canines on both sides showed a similar average root length (16.78 ± 1.94 mm on the right side and 16.54 ± 2.11 mm on the left side), which was longer than the corresponding incisors by 3.2 mm (*p* < 0.0001) (Table 1; Fig. 3A). The mandibular canine, lateral incisor, and central incisor groups exhibited significant pairwise differences in average root length (Fig. 3B). Mandibular canines showed a greater average root length than lateral incisors (with a difference of 2.1 mm) and central incisors (with a difference of 3.3 mm) (*p* < 0.0001) (Table 1; Fig. 3B). Mandibular lateral incisors were also observed to have a significantly greater root length than central incisors, with a difference of 1.2 mm (*p* < 0.0001) (Table 1; Fig. 3B). The data showed that root length on the buccal-lingual side was lower than that on the mesial-distal side (Table 1). 

The CEJ-C was calculated as the subtraction of root lengths, which was elevated in incisor groups in the maxilla (*p* < 0.0001) (Fig. 3C), while no significant difference in the CEJ-C was observed in the mandible (Fig. 3D). Mandibular anterior teeth showed a higher valid rate (V) than maxillary anterior teeth (*p* < 0.001) since some teeth were missing or had a compromised contour. Mandibular teeth were also found to have fewer wedge defects (WDs) concerning the tooth contour integrity than maxillary anterior teeth (*p* < 0.0001) (Table 1; Fig. 3 E–F).

**Table 1:** Root lengths (mm) of maxillary and mandibular anterior teeth

			3^R^		2^R^		1^R^		1^L^		2 ^L^		3 ^L^	
Maxilla	V	%	351	87.5%	336	83.8%	312	77.8%	313	78.1%	338	84.3%	350	87.3%
		BL		15.76 ± 1.91		12.22 ± 1.50		12.11 ± 1.67		12.08 ± 1.75		12.20 ± 1.47		15.59 ± 2.07
		MD		17.88 ± 2.03		14.74 ± 1.67		14.66 ± 1.76		14.56 ± 1.81		14.61 ± 1.61		17.49 ± 2.21
		AL		16.78 ± 1.94		13.48 ± 1.54		13.39 ± 1.69		13.32 ± 1.74		13.40 ± 1.49		16.54 ± 2.11
		C–C		2.00(2.50–1.50)		2.52 ± 0.76		2.55 ± 0.67		2.60 (3.00–2.00)		2.41 ± 0.78		1.90 (2.40–1.40)
	WD	%	13	3.2%	19	4.7%	19	4.7%	19	4.7%	14	3.5%	12	3.0%
Mandible	V	%	375	93.5%	360	90.0%	357	89.0%	362	90.3%	360	90.0%	373	93.0%
		BL		14.45 ± 1.71		12.49 ± 1.28		11.31 ± 1.20		11.24 ± 1.17		12.46 ± 1.24		14.62 ± 1.66
		MD		16.35 ± 1.76		14.32 ± 1.35		13.14 ± 1.28		13.15 ± 1.22		14.25 ± 1.32		16.48 ± 1.80
		AL		15.40 ± 1.70		13.40 ± 1.27		12.22 ± 1.20		12.19 ± 1.16		13.36 ± 1.24		15.55 ± 1.70
		C–C		1.80(2.30–1.50)		1.80 (2.20–1.80)		1.83 ± 0.64		1.88 ± 0.67		1.76 ± 0.68		1.86 ± 0.68
	WD	%	4	1.0%	8	2.0%	9	2.2%	5	1.2%	7	1.7%	4	1.0%

Data expressed as mean ± SD / median (percentile). V: valid; WD: wedge defect; BL: buccal-lingual root length; MD: mesial-distal root length; AL: average root length; C–C: CEJ-C; 1^R/L^: incisor; 2^R/L^: lateral incisor; 3^R/L^: canine; 1/2/3^R^: anterior teeth in the right side; 1/2/3^L^: anterior teeth in the left side. Normally distributed variables were reported as mean ± SD, while medians were used to describe non-normally distributed data

### Root furcation in maxillary and mandibular anterior teeth

**Fig. 4:**
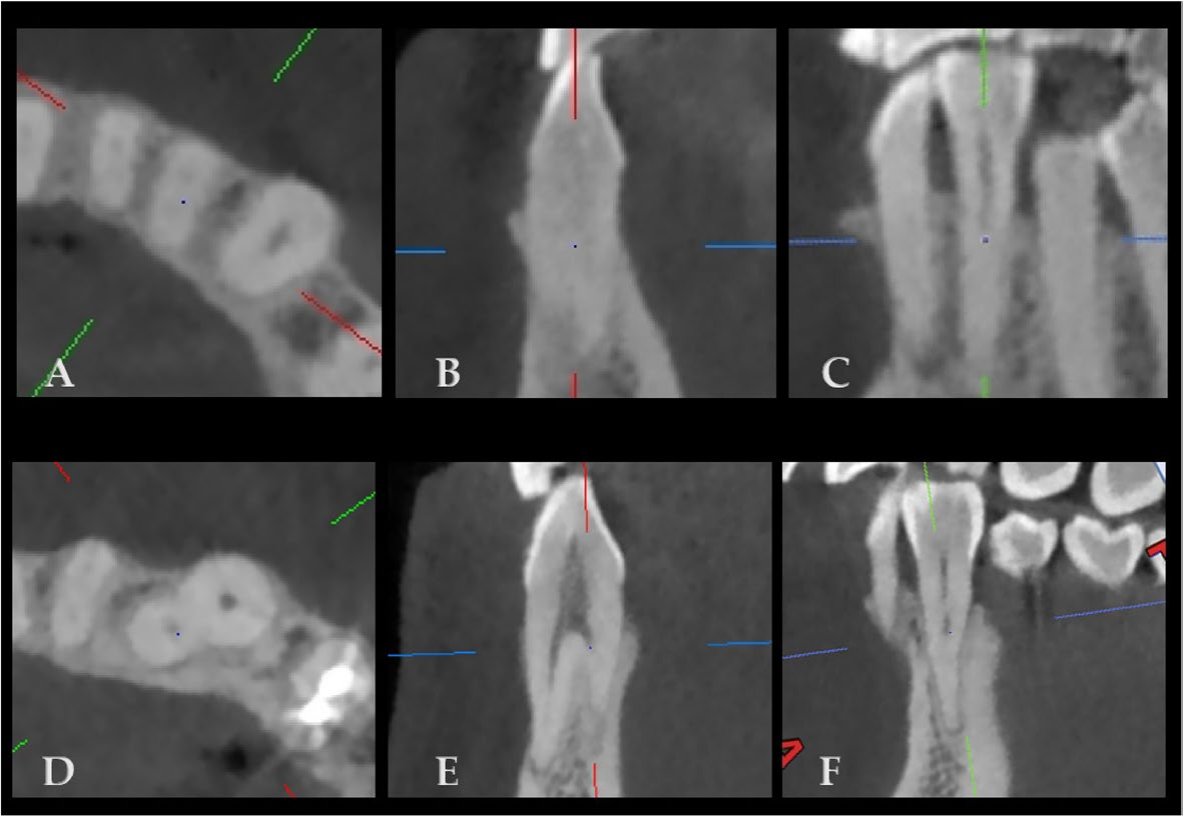
CBCT images of the mandibular lateral incisor and canine with root furcation/two roots in 3D view. (**A**) (**D**) transverse view; (**B**) (**E**) sagittal view; (**C**) (**F**) coronal view. (**A**) (**B**) (**C**) Image of mandibular lateral incisor with root furcation; (**D**) (**E**) (**F**) Image of mandibular canine with root furcation

No root furcation was observed in maxillary anterior teeth. However, mandibular canines and lateral incisors were both observed to have root furcation, while only the central incisor was not detected with root furcation. The frequency of furcation was 1.0% for the right mandibular canine, 1.5% for the left mandibular canine, and 0.2% for both mandibular lateral incisors (Table 2). CBCT schematic diagrams of the bi-root in the mandibular lateral incisor (Fig. 4A-C) and canine (Fig. 4D-F) were shown in Fig. 4.

**Table 2:** Proportion of root furcation in maxillary and mandibular anterior teeth

		3^R^		2^R^		1^R^		1^L^		2^L^		3^L^	
		F	P	F	P	F	P	F	P	F	P	F	P
Maxilla	0	401	100	401	100	401	100	401	100	401	100	401	100
	1	-	-	-	-	-	-	-	-	-	-	-	-
Mandible	0	397	99.0	401	100	401	100	401	100	400	99.8	395	98.5
	1	4	1.0	1	0.2	-	-	-	-	1	0.2	6	1.5

*F* Frequency, *P* Percentage (%), 0: no root furcation; 1: one root furcation; 1 ^R/L^: incisor; 2 ^R/L^: lateral incisor; 3 ^R/L^: canine; 1/2/3^R^: anterior teeth in the right side; 1/2/3^L^: anterior teeth in the left side

### Root canals in maxillary and mandibular anterior teeth

The number of root canals was calculated and analyzed, as shown in Table 3. The mandible was observed with more types of root canals than the maxilla. Only maxillary right central incisors and left lateral incisors were found to have a single root canal, while all other anterior teeth were observed to have various canal types. Among these, the mandible showed a higher percentage of bi-canals than the maxilla.

**Table 3:** Type of root canals in maxillary and mandible anterior teeth

		3^R^		2^R^		1^R^		1^L^		2 ^L^		3 ^L^	
		F	P	F	P	F	P	F	P	F	P	F	P
Maxilla	1–1	361	99.2	352	99.2	331	100	331	99.7	352	100	360	99.4
	2–1	1	0.3	-	-	-	-	1	0.3	-	-	1	0.3
	1–2-1	2	0.5	1	0.3	-	-	-	-	-	-	1	0.3
	2–2	-	-	-	-	-	-	-	-	-	-	-	-
	1–2	-	-	-	-	-	-	-	-	-	-	-	-
	2–1-2	-	-	-	-	-	-	-	-	-	-	-	-
	1–2-1–2	-	-	-	-	-	-	-	-	-	-	-	-
	3–3	-	-	-	-	-	-	-	-	-	-	-	-
	2–1-2–1	-	-	-	-	-	-	-	-	-	-	-	-
	1/O	-	-	2	0.6	-	-	-	-	-	-	-	-
Mandible	1–1	350	93.1	299	81.3	343	93.7	338	92.1	300	81.7	352	94.9
	2–1	3	0.8	7	1.9	4	1.1	3	0.8	8	2.2	7	1.9
	1–2-1	20	5.3	55	14.9	15	4.1	20	5.4	52	14.2	8	2.2
	2–2	-	-	1	0.3	-	-	2	0.5	-	-	-	-
	1–2	-	-	3	0.8	2	0.5	3	0.8	2	0.5	-	-
	2–1-2	-	-	-	-	-	-	-	-	2	0.5	-	-
	1–2-1–2	1	0.3	3	0.8	2	0.5	1	0.3	3	0.8	3	0.8
	3–3	1	0.3	-	-	-	-	-	-	-	-	-	-
	2–1-2–1	1	0.3	-	-	-	-	-	-	-	-	1	0.3
	1/O	-	-	-	-	-	-	-	-	-	-	-	-

*F* Frequency, *P* Percentage (%); 1 ^R/L^: incisor; 2 ^R/L^: lateral incisor; 3 ^R/L^: canine; 1/2/3^R^: anterior teeth in the right side; 1/2/3^L^: anterior teeth in the left side. Type I (1–1); Type II (2–1); Type III (1–2-1); Type IV (2–2); Type V (1–2); Type VI (2–1-2); Type VII (1–2-1–2); Type VIII (3–3); ST I (2–1-2–1); ST II (1/O)

Mandibular lateral incisors were found to have the highest rate of bi-canals, while mandibular central incisors and canines showed a similar rate of bi-canals. However, a single root canal still accounted for the majority of canals, and the only tri-canal observed herein was detected in the right mandibular canine (Table 3).

**Fig. 5:**
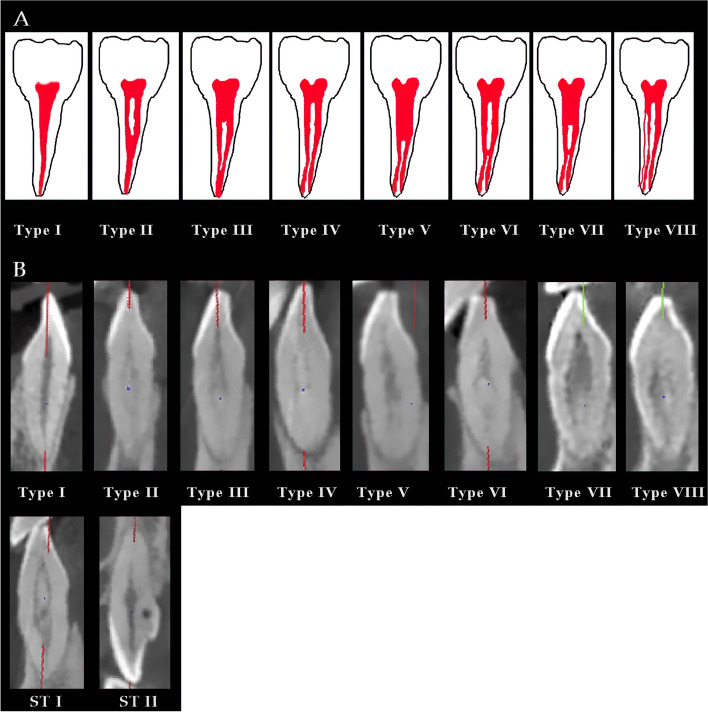
**A** Vertucci’s classification. Type I (1–1); Type II (2–1); Type III (1–2-1); Type IV (2–2); Type V (1–2); Type VI (2–1-2); Type VII (1–2-1–2); Type VIII (3–3). **B** Root canal classification in anterior dentition in the Chinese population. Type I (1–1); Type II (2–1); Type III (1–2-1); Type IV (2–2); Type V (1–2); Type VI (2–1-2); Type VII (1–2-1–2); Type VIII (3–3); ST I (2–1-2–1); ST II (1/O)

The type of root canal was determined according to Vertucci’s classification (Fig. 5A). According to Vertucci’s classification, root canals were classified into eight groups: Type I (1–1); II (2–1); III (1–2-1); IV (2–2); V (1–2); VI (2–1-2); VII (1–2-1–2); and VIII (3–3). Pulp stones were excluded in canal type determination. Among these canal classifications, all eight types were detected in this study. Moreover, two other new types were detected and were referred to as supplementary type I (ST I) and supplementary type II (ST II), as shown in Fig. 5B. ST I represents the 2–1-2–1 root canal type. ST II was a particular type with a small round pulp cavity neighboring the adjacent root canal expressed as 1/O, and this type was observed only in maxillary right lateral incisors with two examples (Fig. 5 and Table 3). However, the variety of canal types in the mandible exceeded those in the maxilla tremendously. We only observed types I, II, III, and ST II in the maxilla, but all types except type ST II could be found in the mandible. Except for type I, type III was the most common type in the mandible, followed by types II and VII. However, type VI and ST II were rarely observed in the mandible (Fig. 5).

## Discussion

Tooth morphology is always a fundamental focus in clinical dentistry, including but not limited to root canal treatment or tooth extraction [22, 23]. Tooth morphology involves various aspects, such as root length, canal variations, and root furcation [24]. There were reports analyzing tooth characteristics within individual nations [11, 13, 25]; however, few studies have focused on a detailed analysis of these characteristics, and thus, it was essential to summarize data within individual nations. Therefore, we aimed to carry out an in-depth investigation of this issue in the Chinese population.

To analyze the root morphology of anterior dentition, we delicately designed a measurement method to ensure it was perpendicular to the field of vision while analyzing CBCT scans (Fig. 1). This was an original design to confirm the accuracy of the measurement, which was never described in detail in previous research [26, 27]. First, the root length was measured as the distance from the CEJ to the root apex. Since the CEJ was not a straight line but an irregular curve at the cervical region of the tooth, the measurement of root length should not start randomly at any point from the CEJ to the root apex. In contrast, the actual root length should theoretically be an average value of every single point at the CEJ to the apex. Therefore, root length was calculated from both the buccal-lingual view and mesial-distal view, and the mean was taken as the root length.

Previous research has reported root lengths in individual countries using CBCT. The average root length of anterior dentition in Brazil was 12 mm in both maxillary and mandibular central incisors [28]. It was reported that for the Korean population, the mean root length of maxillary central incisors was 12.3 mm for males and 11.75 mm for females, and that of maxillary canines was 15.83 mm for males and 15.23 mm for females [29]. Research from India indicated that the average root length of the mandibular central incisor was 12.9 mm, that of the lateral incisor was 12.83 mm, and that of the canine was 14.8 mm [30]. In our research, we found that the root length of maxillary central incisors was 13.3 mm, the root length of the lateral incisors was 13.4 mm, and the root length of the canines was 16.6 mm, while the root length of the mandibular central incisors was 12.2 mm, the root length of the lateral incisors was 13.4 mm, and the root length of the canines was 15.5 mm. We could not compare the root lengths of Chinese to participants of other nationalities based on previous reports because the previous results were greatly influenced by measuring methods, and all related studies have adopted different protocols.

Few studies have examined the CEJ-C. Optical microscopy showed that the CEJ exhibited an irregular and sinuous length in permanent human teeth [31]. It was reported to be dependent on the height of the crown contact area and diameter of the crown labiolingually or buccolingually [32]. Moreover, the CEJ reached maximum curvature in anterior teeth, especially in the interproximal aspect, while keeping a gentler line at posterior teeth [33]. We found that the CEJ-C is tooth location dependent. According to the present research, the CEJ-C was expressed at different levels as the height difference between the buccal-lingual view and mesial-distal view. The CEJ-C was approximately 2.5 mm at incisors and 2.0 mm at canines in the maxilla, which was a statistically significant difference, while the CEJ-C was approximately 1.8 mm with no obvious changes observed among mandibular incisors and canines. Our results seemed to agree with the esthetic requirement that the implant shoulder should be 2 mm above the most apical point of the vestibular CEJ of adjacent teeth [34, 35]. However, it is known that the CEJ-C is an irregular and scalloping curve. The curvature of the CEJ may decrease from mesial to distal, with the greatest curvature observed at the mesial of the central incisor. It may be 1 mm or even greater than that in the distal central incisor [33, 36]. Taken together with our research results, we may conclude that the CEJ-C is approximately 2.5 mm at incisors and 2.0 mm at canines in the maxilla and 1.8 mm at mandibular anterior teeth. This information is important for various aspects of clinical practice. First, the CEJ-C is an essential concept in tooth preparation. A common situation after crown setting is violating biological width interdentally at anterior teeth, which is not aesthetically pleasing and may also be accompanied by bleeding, gingival swelling or even alveolar bone resorption [37]. It is difficult to determine how much tooth preparation would be due to the concealment of the CEJ under the gingival papillae. Therefore, CEJ-C acquaintance is essential during tooth preparation to avoid biological violation. Second, aesthetic restoration is essential for post-core crown preparation or implant design. Due to the extensive damage or even loss of the tooth, cervical design of the new substitution is always a priority. By taking the CEJ-C into consideration, a proper crown for post-core restoration or abutment for implants could aesthetically enhance the final prognosis.

Root furcation is always considered in premolars and molars; however, we also observed furcation in mandibular lateral incisors (0.2%) and canines (1% for right canines and 1.5% for left canines). Previous reports described 0.2% of mandibular canines with bi-roots, but lateral incisors were all single roots in the Malaysian population [11]. A total of 5.2% of permanent mandibular canines were also observed with two roots in the Pakistani population using CBCT [38]. It was also reported that 1.5% of mandibular canines in the Brazilian population and 0.3% of mandibular canines in the Iranian population had double roots according to CBCT [13, 39]. However, the majority of these studies detected bi-root only in mandibular canines, while only a few studies observed lateral incisor furcation. For example, a Chinese group investigated mandibular permanent anterior teeth, finding that 0.3% of mandibular lateral incisors and 0.8% of mandibular canines had double roots [40]. These research results suggested the detection of two roots in anterior dentition was area-dependent. Together with our findings, Chinese patients may have a higher likelihood of root furcation in anterior lateral incisors, which increases the difficulty in clinical diagnosis and further root canal treatment.

Root canal morphology is always a focus in endodontics both clinically and theoretically. To date, there are several canal classification systems. However, dentists still prefer Vertucci’s classification as an important standard [10]. It includes eight subtypes, and even roots in multirooted molars could also be analyzed by this classification. All eight types could be observed in Chinese anterior dentition in this study, and even two more types that could not be included in any group of Vertucci’s classification were also seen. These two supplementary canal types were named ST I (2–1-2–1) and ST II (1/O). It is worth noting that another group examined the Turkish population and observed the 2–1-2–1 canal type, which we mentioned as ST I [41]. We found ST I only in mandibular canines and ST II only in maxillary lateral incisors with rare cases. Moreover, no other reports detected ST II. Therefore, we hypothesize that it may be contributed to a particular tooth development mechanism in maxillary lateral incisors, which needs to be clarified in further research.

Only maxillary right central incisors and left lateral incisors were observed with a single root canal, and all the other teeth in the anterior dentition had double or triple root canals. Therefore, we could infer that the root canal type is not as assumed as bilateral symmetry. Although the mechanism remains unknown, it may be associated with specific growth and development patterns [42]. Interestingly, the triple-root canal in the mandibular canine was found only in one single example. Few documents recording triple canals in the anterior dentition have yet been found, and the only document reporting that was found in the Israeli population [43]. Therefore, the triple-root canal in the anterior dentition is truly sparsely scattered but should still be considered.

Canal types I, II, III, and ST II were observed in the maxilla, while all eight types together with ST I were found in the mandible. Among these, type III accounted for the majority in the mandible, except for type I (Table 3). However, researchers have reported less variety of root canal types in anterior dentition in other populations. The major approaches to detect root canal types were radiography or staining methods. For example, it was reported that Vertucci’s classification types I, II, III, V, and VI were found in Iran's population based on CBCT imaging [13]. Types I, II, III, IV, V, and VI were observed in mandibular anterior teeth among Indian people based on CBCT imaging [30]. The occurrence of two or more root canals in mandibular incisors was as high as 40% in the Israeli population based on CBCT imaging, and Vertucci types I, II, III, IV, V, and VIII were noticed in mandibular incisors [43]. The prevalence rate of bi-root canals was even higher at 47.6% in the Turkish population based on CBCT imaging, and Vertucci types I, II, III, and V, together with 2–1-2–1, were observed in mandibular incisors [41]. In addition to CBCT, other traditional methods were also utilized in determining root canal types. The research group observed extracted mandibular incisors with root canals stained with India ink from the Jordanian population and found that root canal types were type I (73.8%), II (10.9%), III (6.7%), IV (5.1%) and type V (3.6%) [44]. A similar method was utilized to analyze canal types in the northeast Indian population, and Vertucci types II (7.08%), III (22.9%), and V (6.25%) were also observed in addition to type I [45]. Taking these results together, we speculate that the overall trend of root canal variation was much more complicated in the mandible than in the maxilla, and the mandibular canine possessed much more complex variation in canal morphology among anterior dentition. However, it may be area- and race dependent.

These findings together are of great value in clinical dentistry. The results would help improve dental treatment and yield successful outcomes. However, supplements could be made in further studies as follows. First, one limitation of this study is that we took only an initial look into the root morphology of anterior dentition in the Chinese population. Further study is needed to explore the whole dentition in depth on an even larger scale. Second, the CEJ-C seems to be an important concept in clinical dentistry, and we only determined this value for single teeth mesially and distally. We need to compare the length interdentally of two neighboring teeth in further studies. In this way, we may infer the height difference of alveolar bone interdentally according to the CEJ-C. Finally, there are other methods analyzing root canals in addition to Vertucci’s classification [1], and we may need to compare these classifications of canals in the next step to improve the overall research.

## Conclusions

In summary, this study examined the root morphology of anterior dentition in the Chinese population. The average root length of maxillary canines was significantly greater than that of maxillary incisors, and the root lengths of mandibular canines, lateral incisors, and central incisors also showed significant differences. The CEJ-C was markedly reduced in maxillary canines than that in incisors, while the CEJ-C in the mandible remained similar with no significant difference. Root furcations in the mandibular lateral incisors and canines were observed. For root canals, all eight types of Vertucci’s classification were found in this study, while two additional types were observed. Considering the preliminary nature of this study, further in-depth investigation on posterior dentition is needed to broaden the scope of research.

## Data Availability

The data presented in this study are available on request from the corresponding author.

## Abbreviations

CEJ: Cementoenamel junction
CEJ-C: Cementoenamel junction curvature
